# High-throughput sequencing yields a complete mitochondrial genome of *Emberiza godlewskii* (aves, emberidae)

**DOI:** 10.1080/23802359.2023.2247604

**Published:** 2023-08-18

**Authors:** Jiande Li, Lingling Da, Xinkang Bao

**Affiliations:** a College of Life Sciences, Northwest Normal University, Lanzhou, P.R. China; b School of Life Sciences, Lanzhou University, Lanzhou, P.R. China

**Keywords:** *Emberiza godlewskii*, mitochondrial genome, phylogenetic relationship

## Abstract

*Emberiza godlewskii* (Taczanowski, 1874) is a passerine bird of eastern Asia which belongs to the genus *Emberiza* in the bunting family *Emberizidae.* Obtaining the complete mitochondrial genome sequence of *E. godlewskii* is helpful to understand the species delimitation for further study of *E. cia/godlewskii* complex. The circular genome (16,839 bp in length) contains 13 protein-coding genes, 22 transfer RNA genes, 2 ribosomal RNA genes, and 1 control region. The base composition shows that purine percentage (52.87%) is slightly higher than pyrimidine (47.13%). The phylogenetic analysis based on the published data of the mitochondrial genome showed that *E. godlewskii* is closely related to *E. cioides*. This new mitochondrial genome data will provide more essential molecular data for further study related to *E. cia/godlewskii* complex.

## Introduction

The species boundary between *Emberiza. godlewskii* (Taczanowski, 1874) and *E. cia* are more controversial owing to the morphological variation among geographic populations (Vaurie 1956; Mauersberger 1972; Zheng 2005). Our previous research with a small number of gene segments revealed a deep divergence between the southern and northern geographical populations of *E. godlewskii* (Li et al. 2019). Further study on *E. cia/godlewskii* complex with extensive sampling as well as plumage color and morphological measurements demonstrated that the southern subspecies populations form a distinct monophyletic group, while the northern *E. godlewskii* is the sister group of *Emberiza cia*. In other words, there is a distinct southern clade that should be separated from the original *E. godlewskii* (Li et al. 2023). Thus, we recommend the southern *E. godlewskii* subspecies should be recognized as a full species. However, the full mitochondrial genome of *E. godlewskii* is still unknown, which limits this species’ ecological and evolutionary research. In this study, we obtained the complete mitochondrial genome sequence of southern *E. godlewskii* subspecies (*E. godlewskii yunnanensis*) through high-throughput sequencing for the further study on *E. cia/godlewskii* complex.

## Materials and methods

### Ethics statement

The program of sampling collection and experiment in the article has passed the ethical review of Animal and Plant Ethics Committee of Northwest Normal University, and all experiments complied with the guidelines of the committee and the current laws of China.

### Sample collection

The genomic DNA was extracted from the muscle tissue of *E. godlewskii*, which was collected from Jinzhong mountain nature reserve in Guangxi province, China (24.3806˚N 104.5715˚E). A specimen was deposited at the Institute of Zoology and Ecology, College of Life Science, Northwest Normal University (https://sky.nwnu.edu.cn; Dr J. Li, lijd14@nwnu.edu.cn) under the voucher number EG2021017.

### Mitochondrial genome assembly and annotation

Genomic DNA was isolated using a TIANamp Genomic DNA Kit (Tiangen, Beijing, China) according to the manufacturer’s instructions. The complete mitochondrial genome of *E. godlewskii* was sequenced and assembled using Illumina HiSeq 2500 and MitoZ (Meng et al. 2019) respectively. The assembled mitochondrial genome was annotated using the MITOS web server (Bernt et al. 2013) under the invertebrate mitochondrial code.

### Phylogenetic analysis

Sequences used in the phylogenetic analysis were from the NCBI GenBank database and aligned with MAFFT (Katoh and Standley, 2013). The phylogenetic analysis was performed using the IQ-TREE 2 (Minh et al. 2020)

## Results

**Figure 1.**
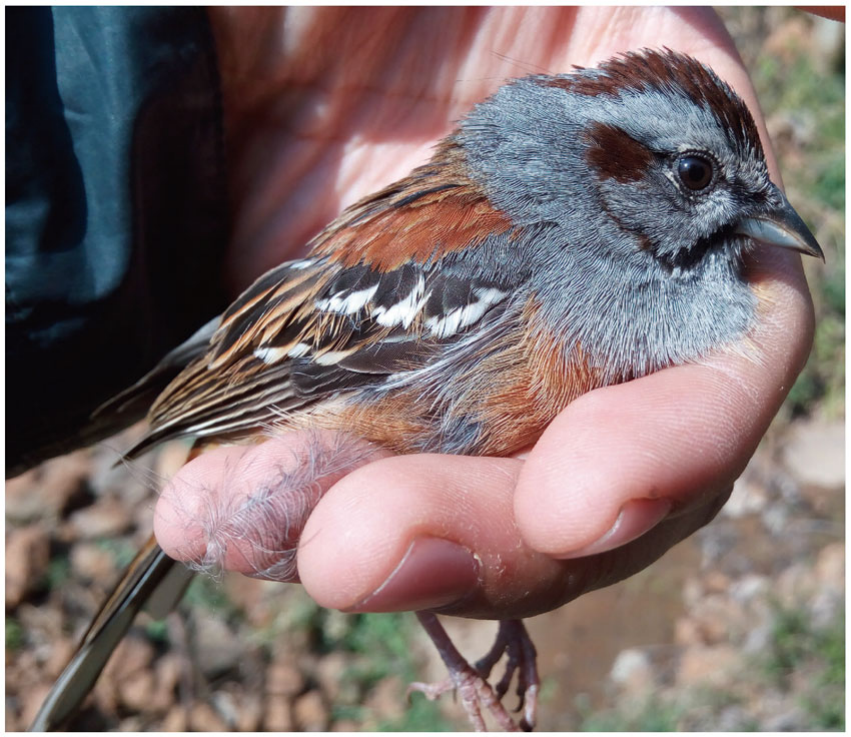
The species image of adult *E. godlewskii*. This bird is characterized by a grey head with chestnut lateral stripe, which is widely distributed in East Asia and tends to select bushy and rocky hill slopes that are often near forests, thickets, ravines, and farm fields. The photograph was taken in Jinzhong mountain nature reserve in Guangxi province of China by Dr X.Bao.

**Figure 2.**
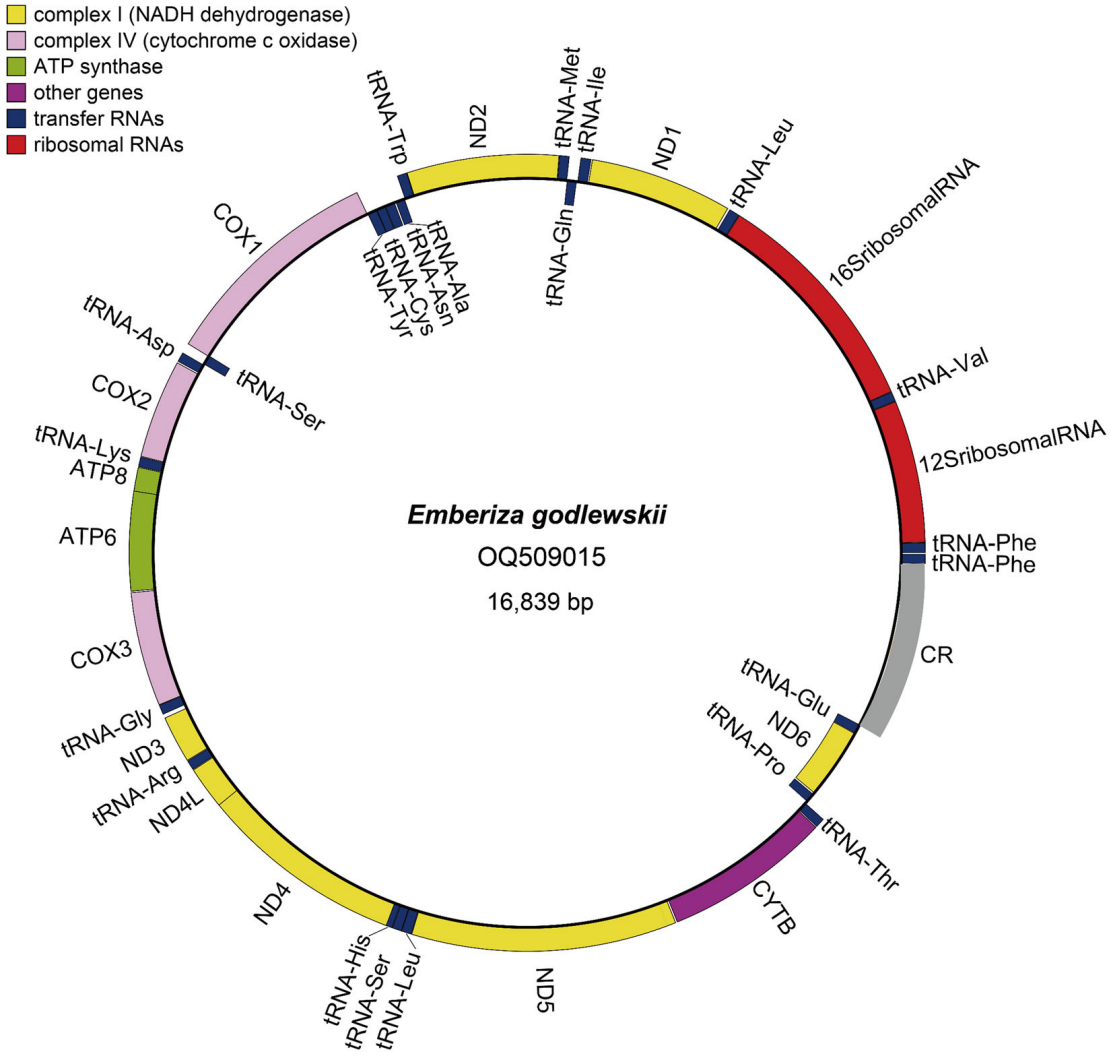
The circular complete mitochondrial genome map of *E. godlewskii.* The complete mtDNA contains 13 PCGs, 23 tRNA genes, 2 rRNA genes (12S rRNA and 16S rRNA), and a control region. The 13 PCGs (ND1, ND2, COX1, COX2, ATP8, ATP6, COX3, ND3, ND4L, ND4, ND5, CYTB and ND6), 23 tRNA genes, and 2 rRNA genes are encoded in the plus and negative strands of the mtDNA. The control region of the mtDNA distributes between genes of tRNA-Glu and tRNA-Phe. The outside of the ring represents the plus strand, while the inside represents the negative strand.

The complete mitochondrial genome of *E. godlewskii* (Figure 1) is circular and its full length is 16,839 bp. The sequence has been deposited in GenBank under accession number OQ509015. The base composition of mitochondrial DNA (mtDNA) shows that the percentage of A + T (52.87%) is slightly higher than G + C (47.13%). The complete mtDNA sequence has 13 protein-coding genes (PCGs), 23 tRNA genes, 2 rRNA genes (12S rRNA and 16S rRNA), and a control region. The 13 PCGs encode ND1, ND2, COX1, COX2, ATP8, ATP6, COX3, ND3, ND4L, ND4, ND5, CYTB and ND6 respectively. In the13 PCGs, eleven PCGs utilize ATG as the start codon, while COX1 translates from GTG and ND3 from ATA; nine PCGs end with complete (TAA: ND2, COX2, ATP8, ATP6, ND3, ND4L, CYTB; TAG: ND6) or incomplete (T: COX3) stop codons, while COX1 and ND1 terminate with AGG and ND4 and ND5 stop with AGA. The 23 tRNA genes range in size from 66 bp encoding tRNA-Ser to 75 bp encoding tRNA-Leu. The 12S and 16S rRNA genes are 976 bp and 1592 bp respectively. The control region of the mtDNA was 1202 bp in length and distributes between genes of tRNA-Glu and tRNA-Phe (Figure 2).

**Figure 3.**
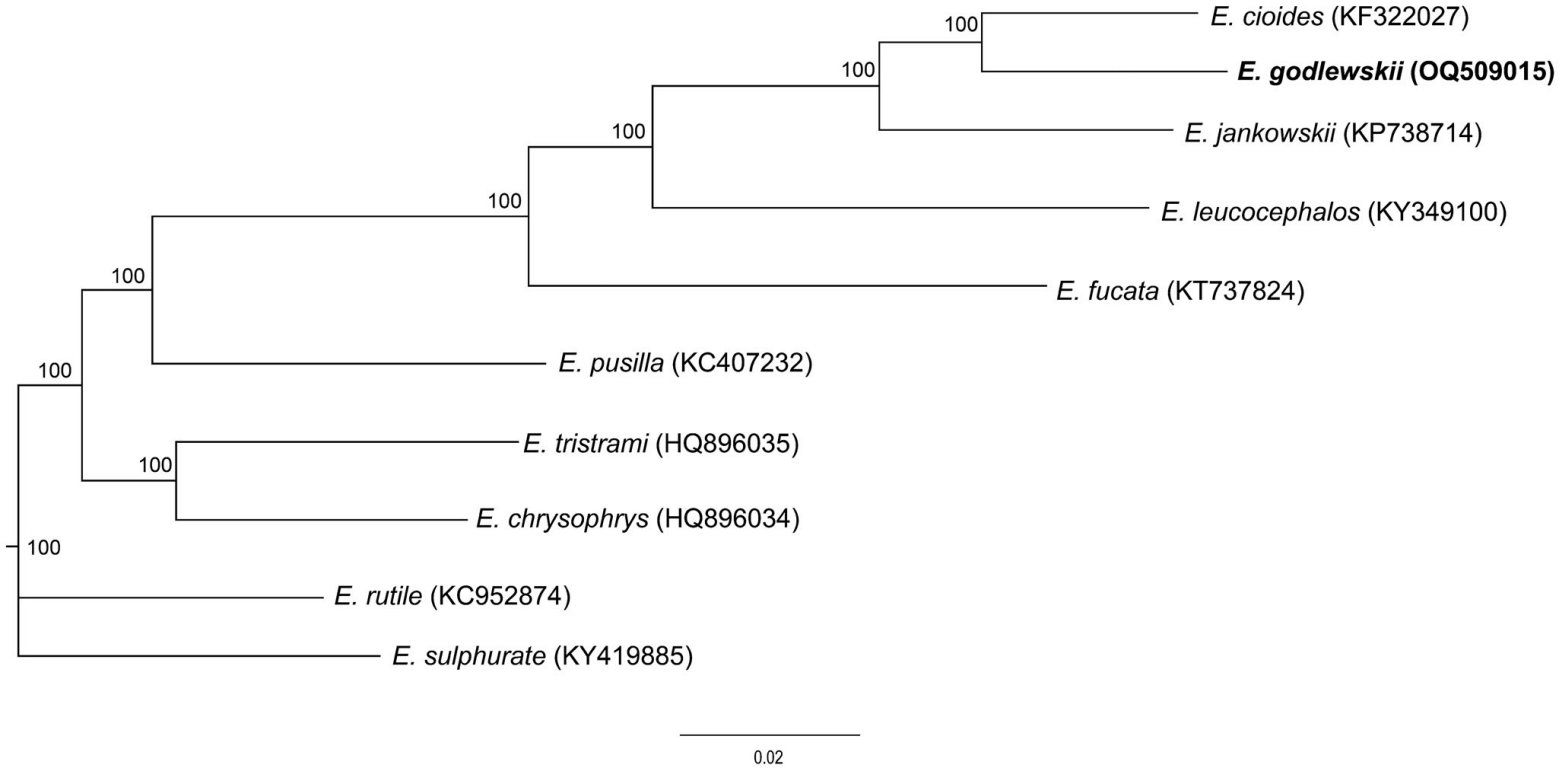
ML tree showing phylogenetic relationship for ten species of genus *Emberiza* based on the concatenated nucleotide sequences of 13 PCGs with HKY + I + G model. Numbers above or below nodes indicated the bootstrap support values estimated with 1000 replicates. The following sequences were used: *Emberiza cioides*, NC_024524, KF322027; *E. jankowskii*, NC_027251, KP738714; *E. leucocephalos*, NC_037692, KY349100; *E. fucata*, NC_033338, KT737824; *E. pusilla*, NC_021408, KC407232; *E. tristrami*, NC_015234, HQ896035; *E. chrysophrys*, NC_015233, HQ896034; *E. rutile*, NC_024925, KC952874; *E. sulfurate*, KY419885. The support values are shown next to the nodes. The species under study is bolded.

To assess the phylogenetic relationships of *E. godlewskii*, we selected other nine species of mitochondrial genomes of genus *Emberiza* birds, which have been published and obtained from GenBank, to construct phylogenetic relationships. We implemented a maximum-likelihood (ML) analysis with the best-fitting model HKY + I + G and 1000 bootstrap replicates. The concatenated nucleotide sequences of 13 PCGs from 10 species of *Emberiza* genus, which included *E. godlewskii,* were used to construct the phylogenetic tree. The phylogenetic analysis showed that *E. godlewskii* is closely related to *E. cioides* based on these available mitochondrial genome data (Figure 3).

## Discussion and conclusion

In summary, the complete mitochondrial genome of *E. godlewskii* is 16,839 bp in length, whose size is almost the same as other genus *Emberiza* birds which have been reported. The phylogenetic relationship based on the published data of mitochondrial genomes demonstrated that *E. godlewskii* is closely related to *E. cioides*. However, the phylogenetic position of *E. godlewskii* is unclear if *E. cia* mitochondrial genome is added. Thus, the mitochondrial genome of *E. godlewskii* reported in this study and sequencing the mitochondrial genome of *E. cia* in the future will provide more essential molecular data for further study related to *E. cia/godlewskii* complex.

## Supplementary Material

Supplemental Material

## Data Availability

The genome sequence data that support the findings of this study are openly available in GenBank at https://www.ncbi.nlm.nih.gov under the accession number OQ509015. The associated BioProject, SRA, and Bio-Sample numbers are PRJNA940058, SRR23680762, and SAMN33550910, respectively.
