# Supplementary figures and images for: High-throughput sequencing yields a complete mitochondrial genome of Emberiza godlewskii (aves, emberidae)
Source: Mitochondrial DNA B Resour. 2023 Aug 18;8(8):882–5. doi: 10.1080/23802359.2023.2247604 (PMC10443981; doi:10.1080/23802359.2023.2247604)

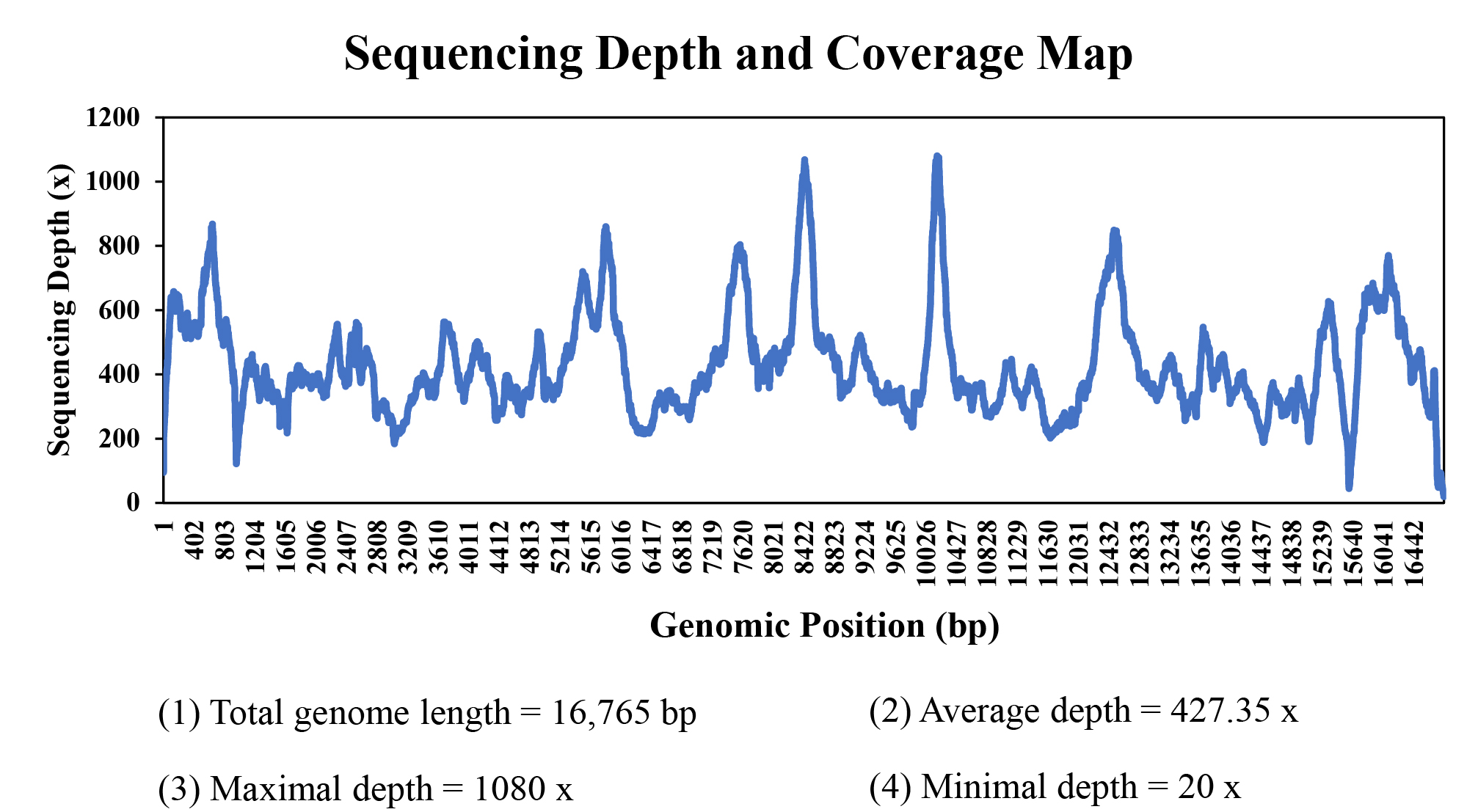

Supplement: Supplemental Material [file TMDN_A_2247604_SM0102.jpg]
